# The characteristics and associations between trait and state time perspective in adolescents with depression: a questionnaire and sandplay study

**DOI:** 10.7717/peerj.18257

**Published:** 2024-10-11

**Authors:** Hanlin Ren, Qing Zhang, Donghuan Rong, Yating Zhang, Yanzhen Ren, Xiaobao Li

**Affiliations:** 1 The Third People’s Hospital of Zhongshan, Zhongshan, China; 2 School of Foreign Studies, Zhongshan Institute, University of Electronic Science and Technology of China, Zhongshan, China; 3 Jinjiang Zimao Central Primary School, Jinjiang, China; 4 Faculty of Education, Henan University, Kaifeng, China

**Keywords:** Adolescence, Depression, Trait time perspective, State time perspective, Nine-grid sandplay therapy

## Abstract

**Background:**

Time perspective is strongly associated with depression. However, the characteristics and associations between trait and state time perspective in adolescents with depression remain unknown.

**Methods:**

A total of 211 adolescents with depression (mean age: 14.60 ± 1.69 years) and 215 healthy controls (mean age: 14.66 ± 1.73 years) were selected and assessed using the Chinese version of the Zimbardo Time Perspective Inventory to quantify their trait time perspective. Thirty adolescents were randomly selected from each group to create nine-grid sandplay productions to assess their state time perspective.

**Results:**

(1) Regarding the trait time perspective, depressed adolescents scored significantly higher on past negative, present fatalistic, present impulsive, and deviation-balanced time perspective (*p* < 0.001) and significantly lower on past positive and future (*p* < 0.05) compared to the control group. (2) In terms of state time perspective, depressed adolescents showed a significantly higher number of squares related to past negative, present negative, and future negative in their nine-grid sandplay productions compared to the control group (*p* < 0.01).Conversely, they had significantly fewer squares associated with past positive, present positive, future positive than the control group (*p* < 0.05). (3) Past negative, present fatalistic, and deviation from balanced time perspective traits were negatively correlated with positive state time perspective and positively correlated with negative state time perspective. Past positive and future traits were positively correlated with positive state time perspective and negatively correlated with negative state time perspective.

**Conclusion:**

Adolescents with depression are characterized by dwelling on the past, having a severely negative attitude toward the past, being pessimistic about the future, and having a tendency to blame fate or external forces for their problems. In the future, standardized methods for measuring state time perspective should be further explored, as well as the effectiveness of the nine-grid sandplay, for improving time perspective in patients with depression.

## Introduction

**Figure 1:**
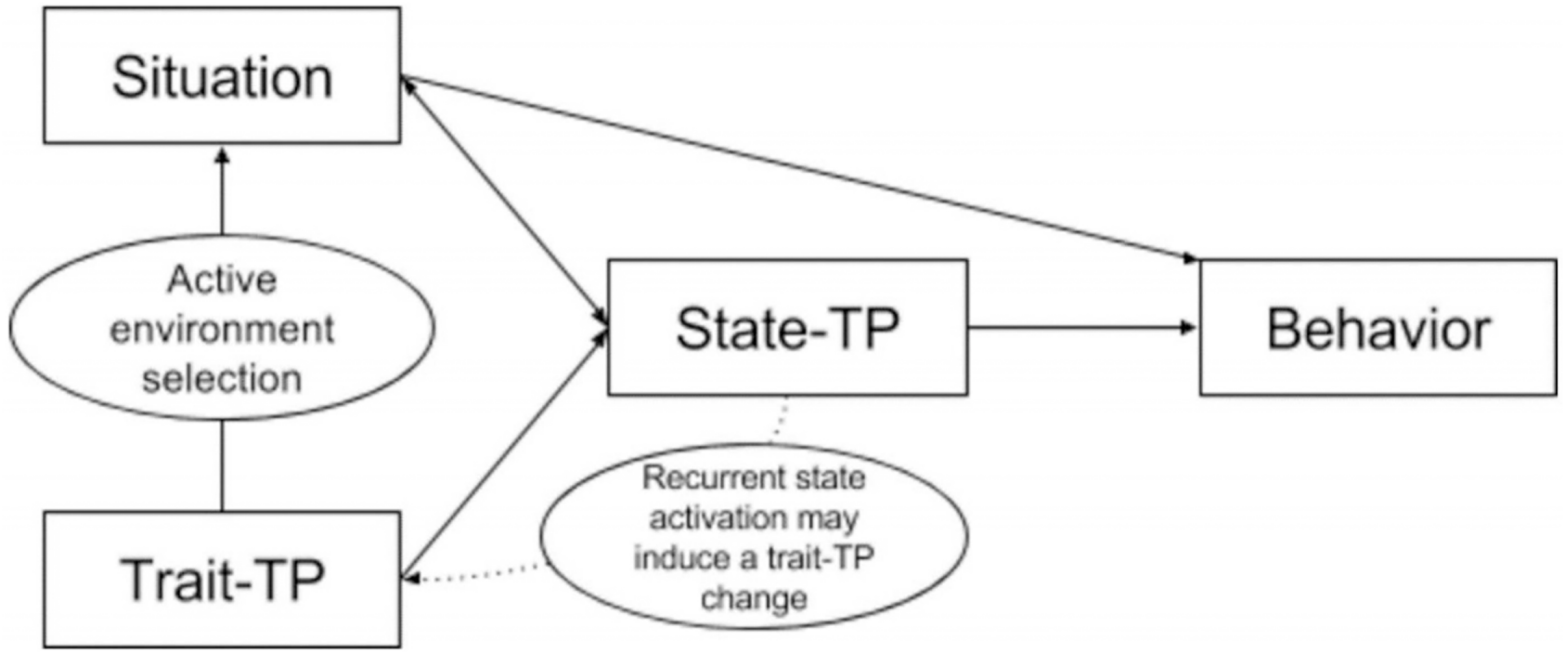
Relationship between trait and state TPs.

Depression is one of the most common mental disorders, affecting 300 million people worldwide (World Health Organization, 2017). It is projected to be the largest global disease burden by 2030, presenting a major public health challenge (Malhi & Mann, 2018). Currently, the etiology of depression remains unclear, and many experts are exploring its pathogenesis from different angles, among which time perspective (TP) is important (Ren et al., 2023). TP can be divided into trait time perspective (trait TP) and state time perspective (state TP). Trait TP is a personality trait referring to an individual’s cognition, time experience, and behavioral tendency concerning past, present, and future (Huang, 2004), as well as functions in the temporal frames of the past, present, and future (Li & Lyu, 2022a; Li, Lyu & Zhang, 2023). State TP is transient concerns and attitudes about the time horizon (past, present, or future) in a given scenario (Stolarski, Fieulaine & Zimbardo, 2018), significantly impacting an individual’s behavior in the present (Klapproth, 2011). Zimbardo categorizes trait TP into past positive (PP; feeling warmth when remembering the past), past negative (PN; holding a negative view of the past), present hedonistic (PH; favoring immediate pleasures without considering the consequences), present fatalistic (PF; feeling helpless when unable to control one’s present life), and future (F; striving for future rewards and goals) (Zimbardo & Boyd, 1999). Zimbardo & Boyd (1999) introduced the idea of balanced time perspective (BTP) defined as “the mental ability to switch effectively among TPs depending on task features, situational considerations, and personal resources, and is an ideal framework for trait TP”. State TP is easily influenced by situations and emotions, *e.g*., environmental uncertainty may reduce an individual’s future orientation and enhance present orientation (Huang, 2004). Individuals under extreme stress may pay more attention to the present (Spiegel, 1997). Negative emotions tend to make people deny the past (Ren et al., 2023), while positive emotions make them future-oriented (Gervey, Igou & Trope, 2005). Moreover, trait and state TPs are closely related. Stolarski, Fieulaine & Zimbardo (2018) created a model of the relationship between the two (Fig. 1). First, trait TP influences behavior by acting on state TP, and activated state TP may also lead to changes in trait TP. Second, trait TP may influence state TP by selecting the environment. Finally, the situation significantly impacts state TP. Thus, when the situation is dominant, such as facing a life threat, the individual’s behavior is mainly determined by the state TP (Klapproth, 2011). When the situation is less stressful, such as choosing a leisure option, behavior mainly responds to the individual’s trait TP (García & Ruiz, 2015). However, human behavior is always determined by state TP, whereas trait TP influences in-the-moment behavior through activated state TP (Stolarski, Fieulaine & Zimbardo, 2018).

Studies showed that trait TP is strongly associated with depression and is a significant predictor of suicide risk (Bitsko et al., 2008; Micillo et al., 2022). The most prominent feature of trait TP in patients with depression is past negativity, with severe pessimism about the past (Zakharova & Trusova, 2019). The second is future negativity, *i.e*., believing in no hope for the future (Hoerger et al., 2012). Another trait is present fatalistic, *i.e*., not believing in the possibility of change (Balashova, 2020; McKay & Cole, 2020) with unbalanced TP (Ren et al., 2023). A few studies indirectly explored the characteristics of state TP in patients with depression and found that depressed patients are characterized by a negative past and a lack of hope for the future (Zhou, 2019). However, these studies mainly focused on adults (Klapproth, 2011; Rush & Grouzet, 2012; Zhou, 2019; Balashova, 2020; McKay & Cole, 2020); hence, whether this is the case for trait and state TP characteristics of adolescents with depression remains unknown.

Adolescence is a critical period to transition from childhood to adulthood and an important turning point in cognitive and emotional development. Compared to children and adults, adolescents are specific in many psychological aspects. For example, Siu et al. (2014) found that compared with adults, adolescents have stronger PH and weaker F traits than adults. Chen et al. (2016) found that adolescents have stronger PN, PP, and PH traits than other age groups and weaker F traits than children and young adults. Therefore, adolescents are characterized by the paradoxical coexistence of strong PN and PP traits, enjoying the present and lacking future goals (Chen et al., 2016). Moreover, adolescents with depression is characterized by a low identification rate, low recovery rate, high suicide rate, and an increasing annual depression incidence compared with adults (Li et al., 2019; Zhang et al., 2023). Current research on state TP in adolescents is lacking. Therefore, comprehensively exploring the specific characteristics of trait and state TPs in adolescents with depression is necessary. This will not only expand the understanding of depression pathogenesis but also will have important practical significance for early intervention and treatment of depression in adolescents.

Questionnaires, projective measures, and behavioral measures are the main methods used to study TP (Lyu & Huang, 2005; Mohammed & Marhefka, 2019). Questionnaires are highly respected for their high reliability and convenience in measuring trait TP, with the most widely used being the Zimbardo Time Perspective Inventory (ZTPI). Due to the variability and instability of state TP (Stolarski, Fieulaine & Zimbardo, 2018), research in this area is more challenging. Therefore, current research on state TP is scarce and lacks a standardized approach. Only a few researchers attempted to explore the characteristics of individual state TP from different perspectives. For example, Klapproth (2011) used emotion induction and temporal discounting tasks to explore the influence of emotion on future decision-making. Individuals in positive mood states are more future-oriented than those in negative mood states. Rush & Grouzet (2012) asked individuals to record their frequency of thinking about the past, present, and future, the degree of pleasure, and the time span for 14 consecutive days, discovering that individual TP changes daily. Zhou (2019) used a nine-grid sandplay to discover that depressed individuals tend to dwell on negative past events and exhibit negative attitudes toward the present and future. However, Klapproth (2011) did not explore the individual’s attitudes toward the past and present in negative/positive emotional states. Rush & Grouzet (2012) and Zhou (2019) did not distinguish between state TP and trait TPs. Those studies mainly explored individuals’ attitudes and perceptions of the past, present, and future in different mental states, which fall under the research category of state TP (Stolarski, Fieulaine & Zimbardo, 2018). Research indicates that sandplay therapy can present an individual’s current psychological state through creation, symbolization, and projection (Stein, 2023). The nine-grid sandplay combines the nine-one drawing method (NOD) with sandplay therapy, presenting an individual’s attitudes and perceptions toward different time intervals (Zhang, 2006). Zhang (2006) used this method to study college students’ allocation of past, present, and future. Based on this, Zhou (2019) explored individuals’ concerns and attitudes toward the past, present, and future. Therefore, the nine-grid sandplay might be a powerful attempt to explore the individual’s state TP.

Depression has become the leading cause of illness and disability in adolescents (Zhang et al., 2023), placing a heavy burden on patients, their families, and society. Research demonstrated that TP is closely related to adolescent depression, which is an important perspective for exploring the development of adolescent depression that provides important insights into depression treatment (Ren et al., 2023). However, a comprehensive understanding of TP characteristics of adolescents with depression is currently lacking. Therefore, the present study aimed to explore the characteristics and relationships between trait and state TPs in adolescents with depression using the Chinese version of ZTPI (ZTPI-C) and nine-grid sandplay, respectively. To our knowledge, this is the first study to comprehensively explore the characteristics of TP in adolescents with depression. Our hypotheses are as follows. (1) Similar to adults with depression (Zhou, 2019; Ren et al., 2023), adolescents with depression are characterized by high PN, high PF, and low F in terms of trait TP. (2) Regarding state TP, they are characterized by past, present, and future negativity and a predominant preoccupation with the past. (3) PN, PF, and deviation-balanced time perspective (DBTP) in trait TP are negatively correlated with positive state TP and positively correlated with negative state TP. (4) PP and F in trait TP are positively correlated with positive state TP and negatively correlated with negative state TP.

## Study 1: characterization of trait tp in adolescents with depression

### Methods

#### Participants

Adolescents with depression were recruited through a psychological outpatient clinic of a specialized psychiatric hospital. The inclusion criteria were (1) patients meeting the Diagnostic and Statistical Manual of Mental Disorders, Fifth Edition (DSM-V) diagnostic criteria for major depressive disorder; (2) patients aged 12–18 years; (3) patients with Self-rating Depression Scale (SDS) score ≥53 (Yuan et al., 2021; Xiao, Xiao & Li, 2023); (4) patients with normal intelligence and hands-on ability; (5) patients without comorbid psychiatric disorders or history of alcohol and drug abuse; (6) patients without apparent non-psychiatric diseases, brain diseases, trauma, or other physical illnesses. A total of 247 questionnaires were distributed, of which 36 questionnaires were excluded due to missing data and irregular responses. Finally, 211 adolescents with depression with a mean age of 14.54 ± 1.62 years were included in this study. Among them, 80 (37.9%) were males, and 131 (62.1%) were females.

The control group was from a middle school. The inclusion criteria comprised (1) people matching with the adolescent depression group in terms of gender, age, and years of education; (2) people not meeting the diagnostic criteria for any of the psychiatric disorders; (3) people scoring <53 on the SDS (Yuan et al., 2021; Xiao, Xiao & Li, 2023); (4) people without a personal history of psychiatric illnesses and family history within three generations; (5) individuals without a history of alcohol/substance abuse; (6) people without a history of craniocerebral trauma or major illnesses. A total of 230 questionnaires were distributed, out of which 15 questionnaires were excluded due to missing data and irregular responses. Finally, 215 healthy adolescents with a mean age of 14.73 ± 1.70 years were included in this study. Of these, 92 (42.8%) were males, and 123 (57.2%) were females.

The study was approved by the ethics committee of the Third People’s Hospital of Zhongshan (NO. SSYLL-2022-01-05). The participants all signed an informed consent form.

#### Tools

The Chinese version of Zimbardo Time Perspective Inventory (ZTPI-C) (Li et al., 2022) was used and included 25 questions with five dimensions (PN, PP, F, PI, and PF, where PI stands for present impulsive). In ZTPI-C, “present hedonistic” was renamed into “present impulsive,” regarding it as the characteristics of impulsivity, carelessness, and disregard for consequences. The ZTPI-C is scored on a five-point scale ranging from 1 (very non-conformant) to 5 (very conformant), with higher scores representing more pronounced TP bias. In this study, Cronbach’s alpha coefficients were 0.75 for PN, 0.73 for PP, 0.67 for F, 0.69 for PI, and 0.62 for PF. The DBTP was used to calculate the balanced TP. The closer the DBTP was to zero, the closer the individual’s TP tended to be balanced in an ideal state (Stolarski, Bitner & Zimbardo, 2011). The following formula was utilized: *DBTP* = $\sqrt {{{\left( {oPN - ePN} \right)}^2} + {{\left( {oPP - ePP} \right)}^2} + {{\left( {oPF - ePF} \right)}^2} + {{\left( {oPH - ePH} \right)}^2} + {{\left( {oF - eF} \right)}^2}}$ where oPN, oPP, oPF, oPH, and oF represent the optimal values of PN, PP, PF, PH, and F scores, respectively, while ePN, ePP, ePF, ePH, and eF are the actual scores for each dimension. According to the revision of Jankowski, Zajenkowski & Stolarski (2020), the critical values for questionnaire dimensions were categorized as 1 for PN, 5 for PP, 5 for F, 1 for PI, and 1 for PF (Li & Lyu, 2022b).

The Self-rating Depression Scale (SDS) was used to assess depressed mood over the past 2 weeks, comprising 20 items on four factors: psychotic affect, somatic disorders, psychomotor disorders, and depressive mental disorders. The tool utilizes a four-point scale, ranging from 1 (no or very little time) to 4 (the vast majority or all of the time). The standardized score is an integer obtained by multiplying the total crude score by 1.25. According to Chinese normative results, a standardized score of ≥53 is considered as having a depressed mood (Yuan et al., 2021; Xiao, Xiao & Li, 2023), which has good applicability in studies of Chinese adolescents (Lu et al., 2023; Xiao, Xiao & Li, 2023).

#### Procedures

Experienced psychiatrists and psychotherapists were the main researchers in this study. Recruited participants were assessed individually or in groups in a hospital or school counseling room. After the participants had answered all the questions, the researcher collected the questionnaires on the spot.

### Results

Independent samples t-tests were used to test the differences between the two groups in depression scores and trait TP scores for each dimension. SDS, PN, PI, PF, and DBTP scores were significantly higher in the adolescent depression group compared to controls (*p* < 0.001), while PP and F scores were significantly lower (*p* < 0.001) (Table 1).

**Table 1:** Test differences between depression and trait TP scores in the two groups.

	Depression group	Control group	*t*	*Cohen’s d*
SDS	70.45 ± 9.13	40.25 ± 7.64	36.97***	3.59
Past negative	4.00 ± 0.62	3.31 ± 0.79	9.98***	0.97
Past positive	2.89 ± 0.91	3.90 ± 0.71	−12.68***	1.23
Present impulsive	3.47 ± 0.79	2.85 ± 0.74	8.28***	0.81
Present fatalistic	3.86 ± 0.82	2.99 ± 0.85	10.61***	1.05
Future	2.75 ± 0.79	3.71 ± 0.72	−13.08***	1.27
DBTP	5.90 ± 1.03	4.19 ± 0.96	17.60***	1.72

**Note:**

*** *p* < 0.001

Paired-sample t-tests revealed that dimension scores of trait TP in adolescents with depression were the highest for PN, followed by PF, PI, PP, and F (*p* < 0.05). Dimension scores of trait TP in controls were the highest for PP, followed by F, PN, PF, and then PI (*p* < 0.05) (Table 2).

**Table 2:** Paired sample t-test for each dimension of trait TP in the two groups.

	Depression group			Control group		
	*M ± SD*	*t*	*Cohen’s d*	*M ± SD*	*t*	*Cohen’s d*
Past negative–past positive	1.10 ± 1.15	13.96***	0.96	−5.88 ± 1.03	−8.35***	5.71
Past negative–present impulsive	0.53 ± 0.89	8.73***	0.60	0.46 ± 0.84	7.94***	0.55
Past negative–present fatalistic	0.15 ± 0.83	2.56*	0.18	0.31 ± 0.95	4.88***	0.33
Past negative–future	1.25 ± 1.08	16.71***	1.16	−0.40 ± 1.05	−5.62***	0.38
Past positive–present impulsive	−0.57 ± 1.20	−6.92***	0.48	1.05 ± 1.01	15.15***	1.04
Past positive–present fatalistic	−0.96 ± 1.31	−10.62***	0.73	0.90 ± 1.12	11.75***	0.80
Past positive–future	0.14 ± 1.04	2.02*	0.13	0.18 ± 0.82	3.30***	0.22
Present impulsive–present fatalistic	−0.39 ± 0.98	−5.75***	0.40	−0.14 ± 0.97	−2.16***	0.14
Present impulsive–future	0.71 ± 1.29	7.98***	0.55	−0.86 ± 1.08	−11.69*	0.80
Present fatalistic–future	1.10 ± 1.31	12.19***	0.84	−0.72 ± 1.12	−9.38***	0.64

**Notes:**

* *p* < 0.05.

*** *p* < 0.001.

Pearson’s correlation was used to analyze the relationship between depression scores and trait TP in both groups. Depression scores in the adolescent depression group were significantly and positively correlated with PN, PF, and DBTP scores (*p* < 0.01) and significantly and negatively correlated with PP and F scores (*p* < 0.01) but non-significantly correlated with PI (*r* = 0.099). Depression scores in the control group were positively correlated with PN and DBTP scores (*p* < 0.05), negatively correlated with PP and F scores (*p* < 0.01), and non-significantly correlated with PI (*r* = 0.004) and PF (*r* = 0.065) (Table 3).

**Table 3:** Correlations between depression and trait TP scores in both groups.

		Past negative	Past positive	Present impulsive	Present fatalistic	Future	DBTP
Depression group	SDS	0.36**	−0.30**	0.09	0.35**	−0.30**	0.47**
Control group	SDS	0.16*	−0.51**	0.01	0.06	−0.39**	0.30**

**Notes:**

* *p* < 0.05.

** *p* < 0.01.

A regression model was constructed with DBTP as the dependent variable and PP, PN, PI, PF, and F as the independent variables, and the regression models for both groups were statistically significant (*F* = 2,268.29, *p* < 0.001 for the depressed group; *F* = 844.82, *p* < 0.001 for the control group). 98.2% of the variance in the dependent variable DBTP in the depressed group could be explained by PP, PN, PI, PF and F (corrected *R*^2 ^= 0.982). Among them PP (*β* = −0.305, *p* < 0.001) and F (*β* = −0.275, *p* < 0.001) negatively influenced DBTP. PN (*β* = 0.333, *p* < 0.001), PI (*β* = 0.322, *p* < 0.001), and PF (*β* = 0.376, *p* < 0.001) positively influenced DBTP. 95.2% of the variation in the dependent variable DBTP in the control group could be explained by PP, PN, PI, PF and F (corrected *R*^*2*^= 0.952). Where PP (*β* = −0.219, *p* < 0.001) and F (*β* = −0.256, *p* < 0.001) had negative effect on DBTP. PN (*β* = 0.451, *p* < 0.00q), PI (*β* = 0.361, *p* < 0.000) and PF (*β* = 0.386, *p* < 0.001) had positive effect on DBTP. In the depressed group, the greatest effect on DBTP was observed in PF, followed by PN, PI, PP, F. In the control group, the greatest effect on DBTP was observed in PN, followed by PF, PI, F, PP.

## Study 2: characteristics of state tp in adolescents with depression

### Methods

#### Participants

From study 1, 30 adolescents with depression and 30 age-matched, education-matched, and gender-matched control adolescents were recruited to participate in the creation of nine-grid sandplay. The adolescent depression group consisted of 12 males (40%) and 18 females (60%) aged 13–17 years (means, 14.7 ± 1.18 years). The control group consisted of 13 males (43.3%) and 17 females (56.7%) aged 13–16 years (mean, 14.4 ± 0.97 years).

#### Tools

The same questionnaires from study 1 were used in study 2.

Regarding the sandplay therapy equipment, the sandbox, with blue walls and the bottom, measured 57 cm × 72 cm × 7 cm. The half of the box was filled with clean sand. A set of various toys was also included: characters, animals, plants, furniture, appliances, buildings, and military equipment. Furthermore, it included a digital camera for recording the sandplay productions made by the client and process-recording article.

#### Process of creating a nine-grid sandplay therapy setup

**Figure 2:**
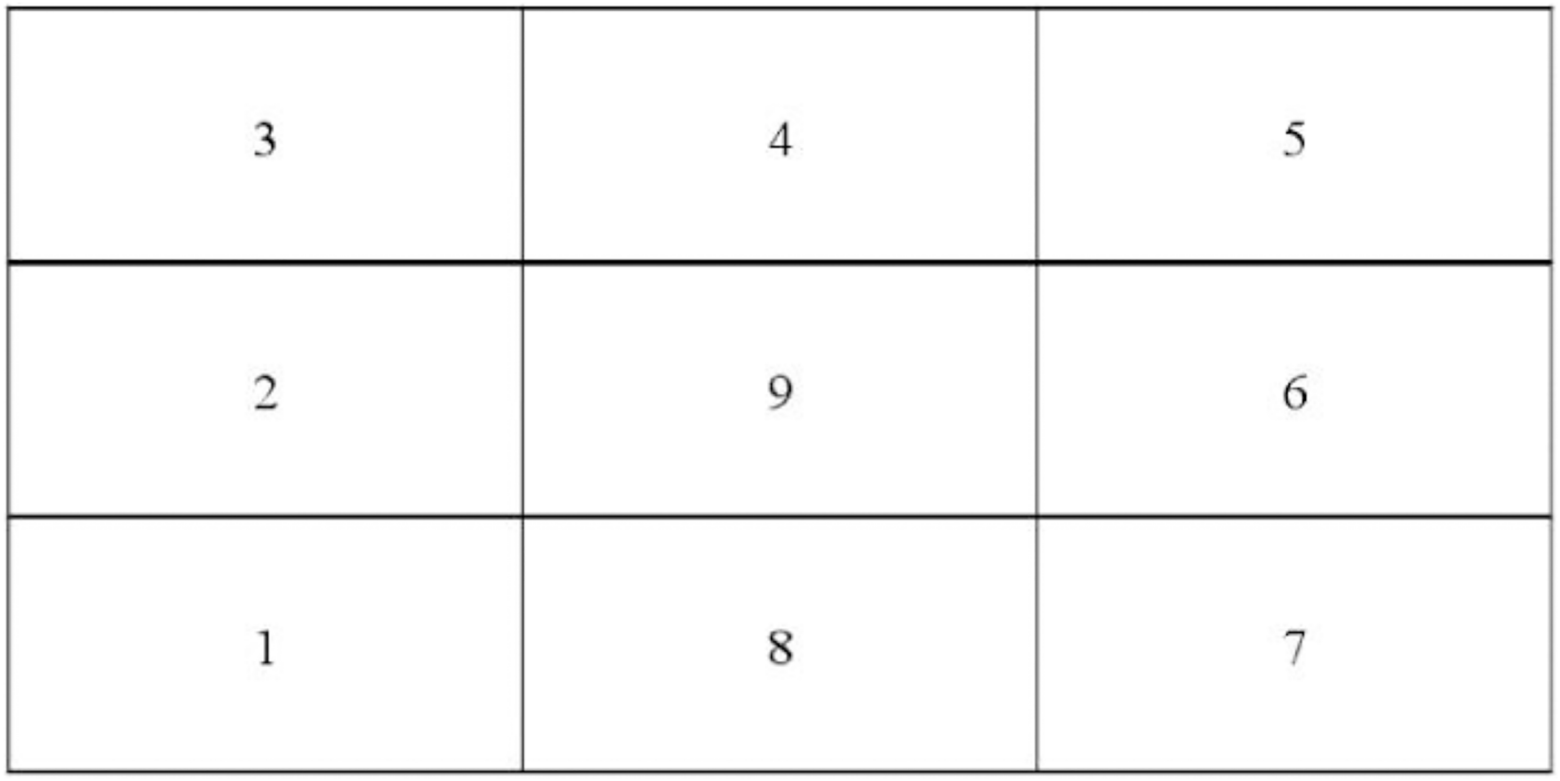
Sequence for creating the nine-grid sandplay layout.

**Figure 3:**
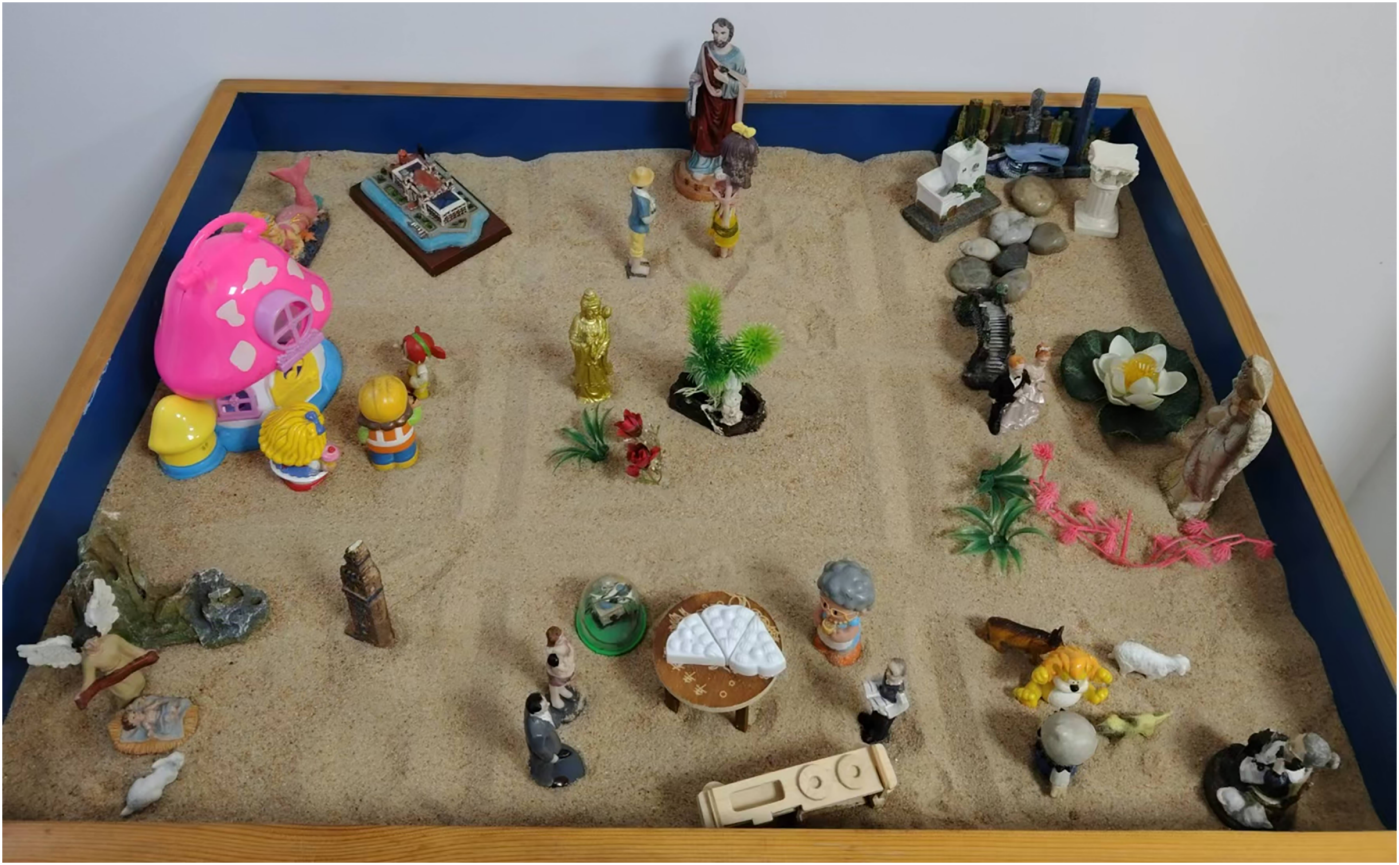
Example of a nine-grid sandplay production.

An experienced psychotherapist or counselor served as the experimenter or research conductor. In a counseling room within a hospital or school, the participant was tasked with creating a nine-grid sandplay creation setup themed around “my whole life” for 50 min. The detailed process is outlined in Table 4. The creation should be freeform as illustrated in Figs. 2 and 3.

**Table 4:** Procedure for creating a nine-grid sandplay.

Content, guidance, and procedures	
Feeling the sand	“Feel how the sand makes you feel.”
Production	“Divide the sandplay into nine relatively uniform cells in a 3 × 3 grid format. Starting from the bottom left corner as cell 1, describe your life in a clockwise direction, ending at the central cell 9. Create a total of nine sandplay scenes and do not skip or leave any cells empty.”
Production experience	“This is your world, please experience it with all your heart.”
Production sharing	“Please describe each of the nine grids as belonging to the past, present, or future, and explain whether the events in each grid are positive or negative.”
Record productions	Record the participants’ descriptions of the nine grids, which can be divided into past negative grid, past positive grid, present negative grid, present positive grid, future negative grid, and future positive grid.
Production demolition	After photographing the sandplay productions for archival purposes, please have the participant dismantle them.

### Results

**Figure 4:**
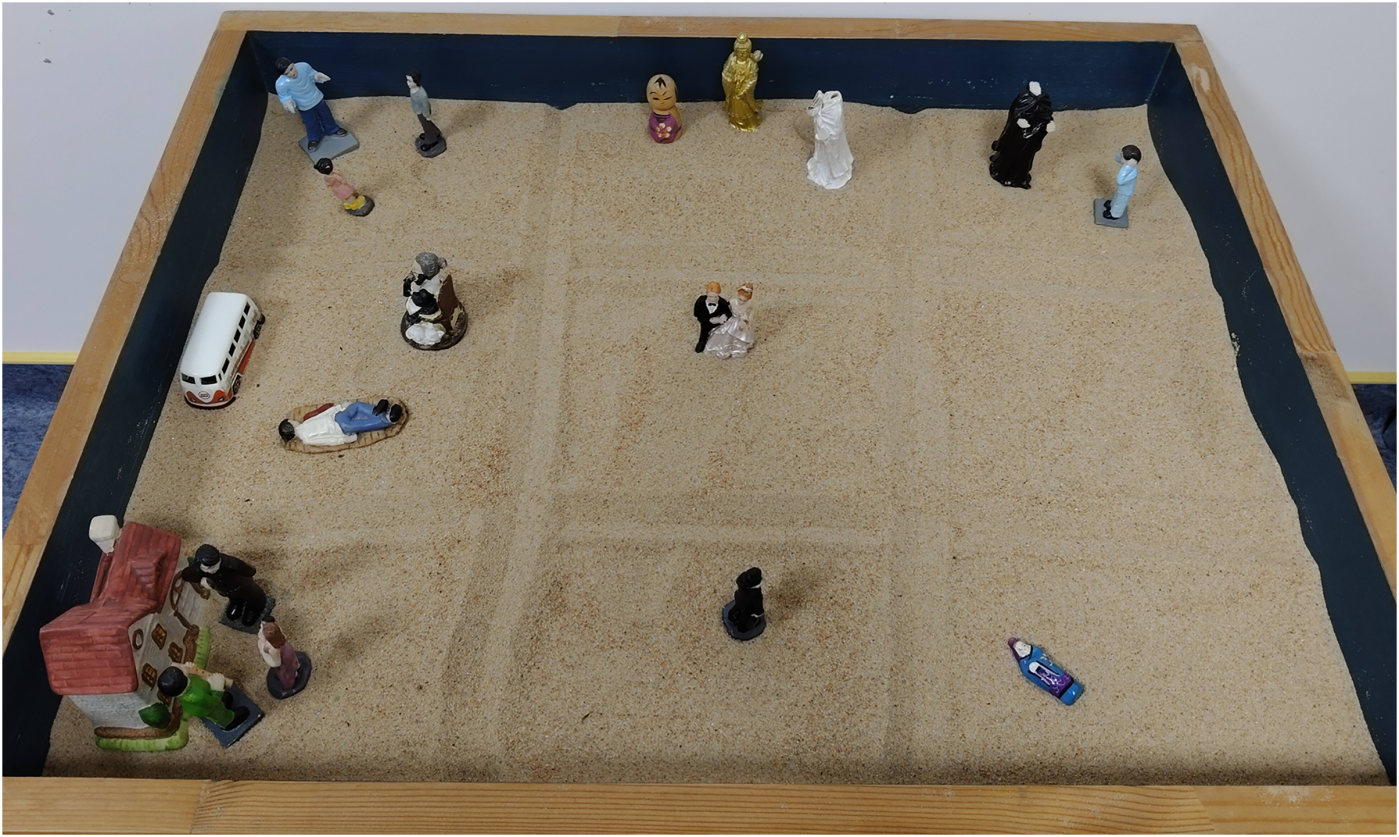
Example of a nine-grid sandplay production for the adolescent depression group.

By recording individuals’ descriptions of each grid, the grids were divided into past negative, past positive, present negative, present positive, future negative, and future positive grids. Figure 4 demonstrates the productions of a senior female student A with depression, who shares the stories of the nine grids. The first grid was the scene of her birth when her father had an affair, her parents fought, and her sister held her. The second grid demonstrated the period when A was in kindergarten, her father was seriously ill and almost died. The third grid was a scene of her parents arguing at home when A was a child. The fourth grid represented the time when A was in junior high school, and a good friend suddenly stopped talking to her. The fifth grid was about a boy in A’s class who always bothers her. The sixth grid was A’s bad state after being harassed. The seventh grid was A’s state improving. The eighth grid was A’s hope for her parents to have a harmonious relationship in the future. The ninth grid was A’s aspiration for the future, hoping to get married and have children. Therefore, after confirming with A, four grids represented the past negative, 0 grids represented the past positive, two grids represented the present negative, one grid represented the present positive, 0 grids represented the future negative, and two grids represented the future positive.

Using an independent samples t-test, the adolescent depression group had significantly more past negative, present negative, future negative, past, and negative mood grids (*p* < 0.01) and fewer past positive, present positive, future positive, present, future, and positive mood grids than the control group (*p* < 0.05), as shown in Table 5.

**Table 5:** Test differences between the two groups in the allocation of the nine-grid sandplay.

	Depressive group	Control group	*t*	*Cohen’s d*
Past negative grids	3.7 ± 1.29	0.37 ± 0.49	13.22***	3.79
Past positive grids	1.07 ± 0.74	2.33 ± 0.95	−5.73***	1.48
Present negative grids	1.30 ± 1.02	0.57 ± 0.81	3.06**	0.79
Present positive grids	0.33 ± 0.47	1.73 ± 0.94	−7.24***	1.88
Future negative grids	1.97 ± 1.42	0.53 ± 0.81	4.77***	1.25
Future positive grids	0.63 ± 0.81	3.47 ± 1.52	−8.98***	2.33
Past grids	4.77 ± 1.52	2.70 ± 0.95	6.29***	1.6
Present grids	1.63 ± 1.18	2.30 ± 0.91	−2.43*	0.64
Future grids	2.60 ± 1.49	4.00 ± 1.33	−3.81***	1.00
Negative mood grids	6.97 ± 1.19	1.47 ± 1.78	14.09***	3.63
Positive mood grids	2.03 ± 1.18	7.53 ± 1.77	−14.09***	3.65

**Notes:**

* *p* < 0.05.

** *p* < 0.01.

*** *p* < 0.001.

**Figure 5:**
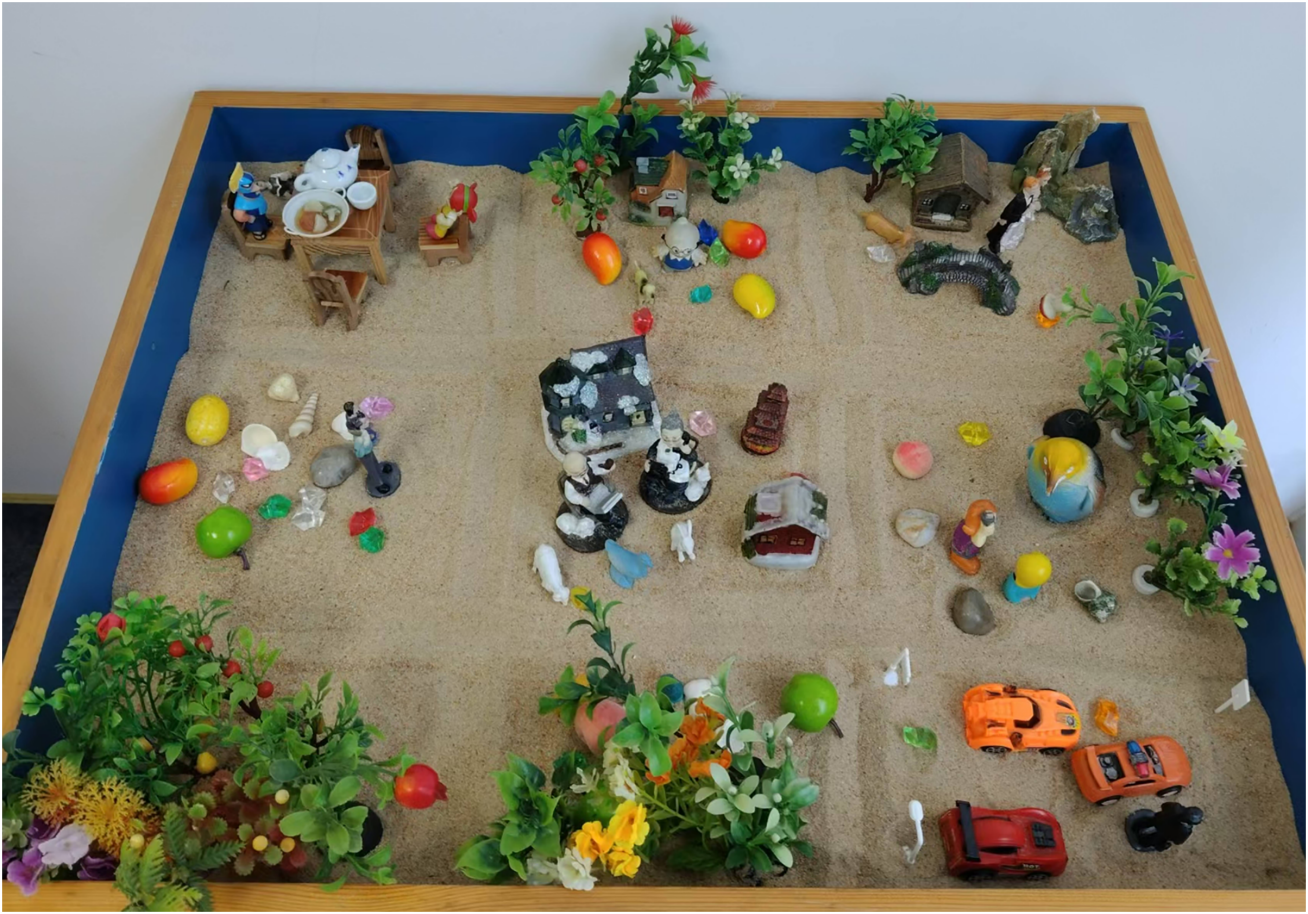
Example of a nine-grid sandplay production for the control group.

Using a paired samples t-test, we examined the allocation of emotions related to time and events in the nine-grid sandplay productions created by the adolescent depression group. The number of grids in each dimension in the adolescent depression group decreased from the highest to the lowest in the following order: past negative grids, future negative grids and present negative grids and past positive grids (the difference between future negative grids and present negative grids was not significant, *p* = 0.055; the difference between present negative grids and past positive grids was not significant, *p* = 0.38; but the difference between future negative grids and past positive grids was significant, *p* < 0.05), and present positive grids and future positive grids (the difference was not significant, *p* = 0.068). The number of grids for each dimension in the control group decreased from the highest to the lowest in the following order: future positive grids, past positive grids, present positive grids, past negative grids, present negative grids, and future negative grids (the difference between the three negative grids was not significant *p* > 0.05) (Table 6). Figures 4 and 5 demonstrate the examples of the two groups’ nine-grid sandplay productions.

**Table 6:** Paired samples t-test for the number of grids in each dimension of nine-grid sandplay productions in the two groups.

	Depressive group			Control group		
	*M ± SD*	*t*	*Cohen’s d*	*M ± SD*	*t*	*Cohens’ d*
Past negative grids–past positive grids	2.63 ± 1.45	9.95***	1.81	−1.97 ± 1.18	−9.06***	1.67
Past negative grids–present negative grids	2.40 ± 2.03	6.48***	1.18	−0.20 ± 0.66	−1.65	0.30
Past negative grids–present positive grids	3.36 ± 1.38	13.39***	2.43	−1.37 ± 1.13	−6.63***	1.21
Past negative grids–future negative grids	1.73 ± 2.32	4.09***	0.75	−0.17 ± 0.79	−1.15	0.22
Past negative grids–future positive grids	3.06 ± 1.64	10.25***	1.87	−3.10 ± 1.84	−9.20***	1.68
Past positive grids–present negative grids	−0.23 ± 1.43	−0.89	0.16	1.77 ± 1.52	6.35***	1.16
Past positive grids–present positive grids	0.73 ± 0.64	6.28***	1.14	0.60 ± 1.16	2.83**	0.52
Past positive grids–future negative grids	−0.90 ± 1.82	−2.7*	0.49	1.80 ± 1.54	6.40***	1.17
Past positive grids–future positive grids	0.43 ± 1.25	1.89	0.34	−1.13 ± 1.94	−3.19**	0.58
Present negative grids–present positive grids	0.97 ± 1.07	4.97***	0.91	−1.17 ± 1.51	−4.23***	0.77
Present negative grids–future negative grids	−0.67 ± 1.83	−2.00	0.37	0.03 ± 0.72	0.25	0.04
Present negative grids–future positive grids	0.67 ± 1.35	2.71*	0.50	−2.90 ± 2.04	−7.78***	1.42
Present positive grids–future negative grids	−1.63 ± 1.79	−4.99***	0.91	1.20 ± 1.42	4.62***	0.85
Present positive grids–future positive grids	−0.3 ± 0.95	−1.72	0.32	−1.73 ± 2.03	−4.67***	0.85
Future negative grids–future positive grids	1.33 ± 1.76	4.13***	0.76	−2.93 ± 2.05	−7.84***	1.43

**Notes:**

* *p* < 0.05.

** *p* < 0.01.

*** *p* < 0.001.

Combining the two datasets, Pearson’s correlation was used to analyze the correlation between the scores of trait TP dimensions and the number of nine-grid sandplay grids (state TP). PN was positively correlated with past negative grids and future negative grids (*ps* < 0.05), negatively correlated with past positive grids and future positive grids (*ps* < 0.01), and not significantly correlated with present negative/positive grids. PP was positively correlated (*ps* < 0.01) with past positive grids, present positive grids, and future positive grids and negatively correlated (*ps* < 0.01) with past negative grids, present negative grids, and future negative grids. PI was positively correlated with past negative grids (*p* < 0.05), negatively correlated with future positive grids (*p* < 0.01), and insignificantly correlated with past positive grids, present negative grids, present positive grids, and future negative grids. PF was positively correlated (*ps* < 0.05) with past negative grids and future negative grids, negatively correlated (*ps* < 0.05) with past positive grids, present positive grids, and future positive grids, and not significantly correlated with present negative grids. F was positively correlated (*ps* < 0.01) with past positive grids, present positive grids, and future positive grids and negatively correlated (*ps* < 0.05) with past negative grids, present negative grids, and future negative grids. DBTP was positively correlated (*ps* < 0.05) with past negative grids, present negative grids, and future negative grids and negatively correlated *(ps* < 0.05) with past positive grids, present positive grids, and future positive grids, as shown in Table 7.

**Table 7:** Correlations between adolescent traits and state TP dimensions.

	Past negative	Past positive	Present impulsive	Present fatalistic	Future	DBTP
Past negative grids	0.323*	−0.634**	0.287*	0.357**	−0.503**	0.525**
Past positive grids	−0.359**	0.497**	−0.187	−0.372**	0.332**	−0.452**
Present negative grids	0.246	−0.368**	0.107	0.192	−0.266*	0.302*
Present positive grids	−0.104	0.520**	−0.147	−0.267*	0.292*	−0.323*
Future negative grids	0.280*	−0.353**	0.228	0.309*	−0.256*	0.375**
Future positive grids	−0.408**	0.541**	−0.333**	−0.338**	0.499**	−0.542**

**Notes:**

* *p* < 0.05.

** *p* < 0.01.

## Discussion

### Characteristics of trait TP in adolescents with depression

The trait TP is potentially important in the diagnosis and treatment of depression (Zakharova & Trusova, 2019; Ren et al., 2023). The present study found that healthy adolescents have warm memories of the past, are hopeful about the future, and believe that they can change their destiny, which is consistent with previous studies (Chen et al., 2016; Worrell & Mello, 2007; Rebiguli-Baikeli, 2021). Healthy adolescents also have high PN experiences, with their PN scores being second only to PP and F scores, which is consistent with previous research (Chen et al., 2016; Rebiguli-Baikeli, 2021).

Compared to healthy adolescents, adolescents with depression have more negative attitudes toward the past, are pessimistic about the future, have more impulsive behavior, and tend to blame fate. This is consistent with the research hypotheses and previous research on adult depression (Balashova, 2020; McKay & Cole, 2020; Ren et al., 2023). Additionally, this study found that depression scores in adolescent depression were positively correlated with PN and PF, negatively correlated with PP and F, and not correlated with PI scores, which is consistent with previous adult studies (Kaya Lefèvre et al., 2019; McKay & Cole, 2020).

Beck (1967) considered the existence of negative evaluations of the self, the environment, and the future in depressed individuals, known as the depressive triad. Researchers believe that the depressive triad is closely related to TP (Zakharova & Trusova, 2019). Negative evaluations of oneself lead to devaluation of past experiences and lower self-esteem in individuals (Zaitseva & Yulia, 2018), and negative attitudes toward the environment reinforce the PF state (Åström et al., 2019). Furthermore, constant preoccupation with negative emotions and symptoms leads to despair about the future (Droit-Volet, 2013). Moreover, brain regions such as the parahippocampal gyrus, precuneus, medial prefrontal cortex, and posterior cingulate cortex play important roles in individual TP (Liu & Feng, 2019; Wu et al., 2019). On the other hand, depressed individuals have abnormal activation in these brain regions, with these morphological changes in neuroplasticity possibly leading to impaired cognitive and affective-emotional processing (Sliz & Hayley, 2012). This leads to long-term negative attitudes toward the past, present, and future.

### Characteristics of state TP in adolescents with depression

State TP is a fleeting concern and attitude about the past, present, or future in a certain scenario (Stolarski, Fieulaine & Zimbardo, 2018). Individuals in different events or moods will have different state TPs (Rush & Grouzet, 2012). A nine-grid sandplay test on the theme of “my whole life” can present an individual’s current perceptions of the past, present, and future. This study found that healthy adolescents were mainly concentrated in the future positive grid. Additionally, there were more positive events than negative events in the grids representing the past and the present. Hence, healthy adolescents have positive state TP, which is consistent with previous findings in adults (Zhou, 2019). When assigning a nine-grid sandplay, adolescents with depression tend to allocate a large number of grids to the past. In the three time grids, negative events outnumber positive events, reflecting their tendencies to dwell on the past, be pessimistic and disappointed about the future (Cai, Zhou & Xu, 2023; Kossewska, Tomaszek & Macałka, 2023), which aligns with their trait TP performance.

The most distinctive characteristic of patients with depression is persistent low mood. According to the associative model of memory and emotion and the theory of spreading activation (Bower & Forgas, 2000), emotions are stored in memory nodes in the form of concepts. Emotion nodes, along with events, contexts, times, and actions related to emotions, collectively form a network as a whole. When an emotional node is activated, emotions and events consistent with its nature are also activated (Bower & Forgas, 2000). Therefore, when adolescents with depression are in a negative emotional state, their memories of negative events are also activated and continuously amplified. Hence, adolescents with depression are often filled with pessimism (Ren et al., 2023). Healthy adolescents are more likely to recall about non-negative emotional events. Therefore, they were not characterized by a high number of negative grids. This demonstrates that the nine-grid sandplay can be a better reflection of an individual’s state TP.

### Relationship between adolescent trait and state TP

This study showed a close relationship between adolescent trait and state TPs, which is in line with the discussions of Stolarski, Fieulaine & Zimbardo (2018). Furthermore, this study explored the relationship between various dimensions of both. First, there was a significant overall correlation between the PP, PF, and F in trait TP and past, present, and future positive/negative in state TP. According to Stolarski, Fieulaine & Zimbardo’s (2018) model, state TP is generally influenced by trait TP. Therefore, when adolescents form a trait TP characterized by high PP, low PF, and high F, they will exhibit more positive emotions when recalling the past, experiencing the present, and envisioning the future. Adolescents with depression formed a trait TP marked by negative past, present fatalistic, and pessimistic future orientation (Ren et al., 2023). Thus, they frequently experience a state of psychological negative pessimism (Lin & Zhang, 2021), manifesting a negative state TP. Second, there was no significant correlation between PN in trait TP and present positive/negative in state TP. On the one hand, this may be related to Chinese culture. The emphasis on hard study and on “no pains, no gains” may have weakened the influence of past negative experiences on present negative state (Jia & Li, 2010). On the other hand, this may be related to the unique stage that adolescents go through as they transition from childhood to adulthood, which is a period of change and uncertainty that affects their TP (Chen et al., 2016). Sample size and cross-cultural studies should be expanded in the future to further clarify the relationship between the two.Third, this study found a significant correlation between PI in trait TP and past negative and future positive in state TP, which might be related to the characteristics of PI. PI refers to an individual’s impulsive, careless, and consequence-ignoring traits (Li et al., 2022). Research shows that individuals with high impulsivity have more negative emotions and are more likely to activate past negative experiences (Yang et al., 2023). Moreover, individuals with high impulsivity tend to choose smaller immediate rewards over larger delayed rewards (Peng et al., 2021), possibly causing a lack of planning for the future (Fite et al., 2008). Additionally, individuals with high impulsivity lack consideration of the negative impacts of behavior on both the present and the future (Fite et al., 2008). This might be one of the reasons why PI is not significantly related to present positive/negative and future negative in the state TP.

## Implications, limitations, and future directions

Adolescence is a key period in which an individual’s personality gradually forms and becomes more stable. By gaining a comprehensive understanding of the characteristics of adolescent TP, we can lay a foundation for exploring the development of TP across an individual’s lifespan. Moreover, we can provide theoretical support for the promotion of adolescent mental health. Since trait TP is a stable personality trait that is difficult to change directly, may be possible to adjust state TP to foster the development of a balanced trait TP in adolescents with depression. Furthermore, sandplay therapy is a psychotherapeutic method that can effectively improve negative mood and promote healthy personality development (Lin & Zhang, 2021). Therefore, the nine-grid sandplay might have an important role in improving depressed mood and promoting the balance of TP in patients with depression, which should be further verified in the future.

This study also has some limitations. First, depression can be classified according to its severity into mild, moderate, and severe forms, as well as into first-episode and recurrent depression (Ren et al., 2023). Adolescents with different types of depression might exhibit different TP. However, this study did not categorize and compare them. Future research is needed to explore the TP characteristics of different adolescent depression subtypes, which is important for the development of targeted treatment plans. Second, the nine-grid sandplay measurement of state TP is a non-standardized method and might have certain issues with reliability and validity. In the future, it is necessary to investigate the standard procedure for measuring state TP using the nine-grid sandplay, as well as the development of diverse standardized methods for measuring state TP, is needed. Lastly, the study had a small sample size for the nine-grid sandplay construction, which might have influenced the results. Future studies should increase the sample size to further explore the characteristics of state TP in adolescents with depression and its relationship with trait TP.

## Conclusions

This study provides the following conclusions. (1) Regarding the trait TP, adolescents with depression are characterized by high PN, high PF, high PI, low PP and low F. (2) Regarding the state TP, adolescents with depression are characterized by a tendency to recall negative past events and to be pessimistic and despondent about the present and future. (3) Adolescent trait and state TPs are closely related.

## Supplemental Information

10.7717/peerj.18257/supp-1 Supplemental Information 1 Raw data and codebook.

10.7717/peerj.18257/supp-2 Supplemental Information 2 STROBE checklist.

## Additional Information and Declarations

### Data Availability

The following information was supplied regarding data availability: The raw measurements are available in the Supplemental File.
