# Supplementary material for: The characteristics and associations between trait and state time perspective in adolescents with depression: a questionnaire and sandplay study
Source: PeerJ. 2024 Oct 11;12:e18257. doi: 10.7717/peerj.18257 (PMC11472794; doi:10.7717/peerj.18257)
Supplement: Supplemental Information 2 [file peerj-12-18257-s002.docx]

STROBE Statement—checklist of items that should be included in reports of observational studies

|  | Item No. | Recommendation | Page  No. | Relevant text from manuscript |
| --- | --- | --- | --- | --- |
| **Title and abstract** | 1 | (*a*) Indicate the study’s design with a commonly used term in the title or the abstract | Page 1 | The Characteristics and Associations Between Trait and State Time Perspective in Adolescents with Depression |
|  |  | (*b*) Provide in the abstract an informative and balanced summary of what was done and what was found | Page 1 | Background, Methods, Results, Conclusion:Adolescents with depression are characterized by dwelling on the past, having a severely negative attitude toward the past, being pessimistic about the future, and having a tendency to blame fate or external forces for their problems |
| Introduction | | | |  |
| Background/rationale | 2 | Explain the scientific background and rationale for the investigation being reported | Page 1 | Time perspective is strongly associated with depression. However, the characteristics and associations between trait and state time perspective in adolescents with depression remain unknown. |
| Objectives | 3 | State specific objectives, including any prespecified hypotheses | Page 4 | To our knowledge, this is the first study to comprehensively explore the characteristics of TP in adolescents with depression.  Our hypotheses are as follows. (1) Similar to adults with depression (Zhou, 2019; Ren et al., 2023), adolescents with depression are characterized by high PN, high PF, and low F in terms of trait TP. (2) Regarding state TP, they are characterized by past, present, and future negativity and a predominant preoccupation with the past. (3) PN, PF, and deviation-balanced time perspective (DBTP) in trait TP are negatively correlated with positive state TP and positively correlated with negative state TP. (4) PP and F in trait TP are positively correlated with positive state TP and negatively correlated with negative state TP. |
| Methods | | | |  |
| Study design | 4 | Present key elements of study design early in the paper | Page 4 | Adolescents with depression were recruited through a psychological outpatient clinic of a specialized psychiatric hospital. A total of 247 questionnaires were distributed, of which 36 questionnaires were excluded due to missing data and irregular responses. Finally, 211 adolescents with depression with a mean age of 14.54 ± 1.62 years were included in this study. Among them, 80 (37.9%) were males, and 131 (62.1%) were females. |
| Setting | 5 | Describe the setting, locations, and relevant dates, including periods of recruitment, exposure, follow-up, and data collection | Page 4 | Adolescents with depression were recruited through a psychological outpatient clinic of a specialized psychiatric hospital.  The control group was from a middle school.The inclusion criteria comprised (1) people matching with the adolescent depression group in terms of gender, age, and years of education; |
| Participants | 6 | (*a*) *Cohort study*—Give the eligibility criteria, and the sources and methods of selection of participants. Describe methods of follow-up  *Case-control study*—Give the eligibility criteria, and the sources and methods of case ascertainment and control selection. Give the rationale for the choice of cases and controls  *Cross-sectional study*—Give the eligibility criteria, and the sources and methods of selection of participants | Page 4 | Adolescents with depression were recruited through a psychological outpatient clinic of a specialized psychiatric hospital.The inclusion criteria were (1) patients meeting the Diagnostic and Statistical Manual of Mental Disorders, Fifth Edition (DSM-V) diagnostic criteria for major depressive disorder; (2) patients aged 12–18 years; (3) patients with Self-rating Depression Scale (SDS) score ≥5...  The control group was from a middle school......... |
|  |  | (*b*) *Cohort study*—For matched studies, give matching criteria and number of exposed and unexposed  *Case-control study*—For matched studies, give matching criteria and the number of controls per case | Page 5 | 211 adolescents with depression with a mean age of 14.54 ± 1.62 years were included in this study. Among them, 80 (37.9%) were males, and 131 (62.1%) were females.  The control group was from a middle school. The inclusion criteria comprised people matching with the adolescent depression group in terms of gender, age, and years of education;  Finally, 215 healthy adolescents with a mean age of 14.73 ± 1.70 years were included in this study. Of these, 92 (42.8%) were males, and 123 (57.2%) were females. |
| Variables | 7 | Clearly define all outcomes, exposures, predictors, potential confounders, and effect modifiers. Give diagnostic criteria, if applicable | Page 5 | 215 healthy adolescents with a mean age of 14.73 ± 1.70 years were included in this study. Of these, 92 (42.8%) were males, and 123 (57.2%) were females.  The Chinese version of Zimbardo Time Perspective Inventory (ZTPI-C) (Li et al., 2022) was used |
| Data sources/ measurement | 8* | For each variable of interest, give sources of data and details of methods of assessment (measurement). Describe comparability of assessment methods if there is more than one group | Page 5 | The Chinese version of Zimbardo Time Perspective Inventory (ZTPI-C) (Li et al., 2022) was used and included 25 questions with 5 dimensions (PN, PP, F, PI, and PF, where PI stands for present impulsive). In ZTPI-C, “present hedonistic” was renamed into “present impulsive,” regarding it as the characteristics of impulsivity, carelessness, and disregard for consequences. The ZTPI-C is scored on a 5-point scale ranging from 1 (very non-conformant) to 5 (very conformant), with higher scores representing more pronounced TP bias. In this study, Cronbach's alpha coefficients were 0.75 for PN, 0.73 for PP, 0.67 for F, 0.69 for PI, and 0.62 for PF |
| Bias | 9 | Describe any efforts to address potential sources of bias | Page 4,6 | The inclusion criteria comprised (1) people matching with the adolescent depression group in terms of gender, age, and years of education;  Experienced psychiatrists and psychotherapists were the main researchers in this study. |
| Study size | 10 | Explain how the study size was arrived at | Page 4 | Adolescents with depression were recruited through a psychological outpatient clinic of a specialized psychiatric hospital. A total of 247 questionnaires were distributed, of which 36 questionnaires were excluded due to missing data and irregular responses |

Continued on next page

| Quantitative variables | 11 | Explain how quantitative variables were handled in the analyses. If applicable, describe which groupings were chosen and why | Page 6 | Measures describing the normal distribution are presented using M ± SD |
| --- | --- | --- | --- | --- |
| Statistical methods | 12 | (*a*) Describe all statistical methods, including those used to control for confounding | Page 6 | Independent samples t-tests were used to test the differences between the two groups in depression scores and trait TP scores for each dimension |
|  |  | (*b*) Describe any methods used to examine subgroups and interactions | Page 6 | Pearson's correlation was used to analyze the relationship between depression scores and trait TP in both groups. |
|  |  | (*c*) Explain how missing data were addressed | Page 4 | of which 36 questionnaires were excluded due to missing data and irregular responses |
|  |  | (*d*) *Cohort study*—If applicable, explain how loss to follow-up was addressed  *Case-control study*—If applicable, explain how matching of cases and controls was addressed  *Cross-sectional study*—If applicable, describe analytical methods taking account of sampling strategy | Page 4 | The control group was from a middle school. The inclusion criteria comprised (1) people matching with the adolescent depression group in terms of gender, age, and years of education |
|  |  | (*e*) Describe any sensitivity analyses |  |  |
| Results | | | | |
| Participants | 13* | (a) Report numbers of individuals at each stage of study—eg numbers potentially eligible, examined for eligibility, confirmed eligible, included in the study, completing follow-up, and analysed | Page 4 | A total of 247 questionnaires were distributed, of which 36 questionnaires were excluded due to missing data and irregular responses. Finally, 211 adolescents with depression with a mean age of 14.54 ± 1.62 years were included in this study. Among them, 80 (37.9%) were males, and 131 (62.1%) were females.  A total of 230 questionnaires were distributed, out of which 15 questionnaires were excluded due to missing data and irregular responses. Finally, 215 healthy adolescents with a mean age of 14.73 ± 1.70 years were included in this study. Of these, 92 (42.8%) were males, and 123 (57.2%) were females. |
|  |  | (b) Give reasons for non-participation at each stage | Page 4 | which 15 questionnaires were excluded due to missing data and irregular responses |
|  |  | (c) Consider use of a flow diagram |  |  |
| Descriptive data | 14* | (a) Give characteristics of study participants (eg demographic, clinical, social) and information on exposures and potential confounders | Page 4 | 211 adolescents with depression with a mean age of 14.54 ± 1.62 years were included in this study. Among them, 80 (37.9%) were males, and 131 (62.1%) were females.  215 healthy adolescents with a mean age of 14.73 ± 1.70 years were included in this study. Of these, 92 (42.8%) were males, and 123 (57.2%) were females. |
|  |  | (b) Indicate number of participants with missing data for each variable of interest | Page 4 | A total of 247 questionnaires were distributed, of which 36 questionnaires were excluded due to missing data and irregular responses  A total of 230 questionnaires were distributed, out of which 15 questionnaires were excluded due to missing data and irregular responses |
|  |  | (c) *Cohort study*—Summarise follow-up time (eg, average and total amount) |  |  |
| Outcome data | 15* | *Cohort study*—Report numbers of outcome events or summary measures over time |  |  |
|  |  | *Case-control study—*Report numbers in each exposure category, or summary measures of exposure | Page 6 | Independent samples t-tests were used to test the differences between the two groups in depression scores and trait TP scores for each dimension. SDS, PN, PI, PF, and DBTP scores were significantly higher in the adolescent depression group compared to controls (*p* < 0.001), while PP and F scores were significantly lower (*p* < 0.001) (Table 1).  Paired-sample t-tests revealed that dimension scores of trait TP in adolescents with depression were the highest for PN, followed by PF, PI, PP, and F (*p* < 0.05). Dimension scores of trait TP in controls were the highest for PP, followed by F, PN, PF, and then PI (*p* < 0.05) (Table 2) |
|  |  | *Cross-sectional study—*Report numbers of outcome events or summary measures |  |  |
| Main results | 16 | (*a*) Give unadjusted estimates and, if applicable, confounder-adjusted estimates and their precision (eg, 95% confidence interval). Make clear which confounders were adjusted for and why they were included | Page 6 | Independent samples t-tests were used to test the differences between the two groups in depression scores and trait TP scores for each dimension. SDS, PN, PI, PF, and DBTP scores were significantly higher in the adolescent depression group compared to controls (*p* < 0.001), while PP and F scores were significantly lower (*p* < 0.001) (Table 1).  Paired-sample t-tests revealed that dimension scores of trait TP in adolescents with depression were the highest for PN, followed by PF, PI, PP, and F (*p* < 0.05). Dimension scores of trait TP in controls were the highest for PP, followed by F, PN, PF, and then PI (*p* < 0.05) (Table 2)  Pearson's correlation was used to analyze the relationship between depression scores and trait TP in both groups. |
|  |  | (*b*) Report category boundaries when continuous variables were categorized | Page 4 | patients with Self-rating Depression Scale (SDS) score ≥53 |
|  |  | (*c*) If relevant, consider translating estimates of relative risk into absolute risk for a meaningful time period |  |  |

Continued on next page

| Other analyses | 17 | Report other analyses done—eg analyses of subgroups and interactions, and sensitivity analyses |  |  |
| --- | --- | --- | --- | --- |
| Discussion | | | | |
| Key results | 18 | Summarise key results with reference to study objectives | Page 12 | This study provides the following conclusions. (1) Regarding the trait TP, adolescents with depression are characterized by high PN, high PF, high PI, low PP and low F. (2) Regarding the state TP, adolescents with depression are characterized by a tendency to recall negative past events and to be pessimistic and despondent about the present and future. (3) Adolescent trait and state TPs are closely related |
| Limitations | 19 | Discuss limitations of the study, taking into account sources of potential bias or imprecision. Discuss both direction and magnitude of any potential bias | Page 11 | Lastly, the study had a small sample size for the nine-grid sandplay construction, which might have influenced the results. Future studies should increase the sample size to further explore the characteristics of state TP in adolescents with depression and its relationship with trait TP. |
| Interpretation | 20 | Give a cautious overall interpretation of results considering objectives, limitations, multiplicity of analyses, results from similar studies, and other relevant evidence | Page 10 | According to the associative model of memory and emotion and the theory of spreading activation, emotions are stored in memory nodes in the form of concepts. Emotion nodes, along with events, contexts, times, and actions related to emotions, collectively form a network as a whole. When an emotional node is activated, emotions and events consistent with its nature are also activated (Bower & Forgas, 2000). Therefore, when adolescents with depression are in a negative emotional state, their memories of negative events are also activated and continuously amplified. Hence, adolescents with depression are always filled with pessimism |
| Generalisability | 21 | Discuss the generalisability (external validity) of the study results | Page 11 | Adolescence is a key period in which an individual’s personality gradually forms and becomes more stable. By gaining a comprehensive understanding of the characteristics of adolescent TP, we can lay a foundation for exploring the development of TP across an individual’s lifespan. Moreover, we can provide theoretical support for the promotion of adolescent mental health |
| Other information | |  | | |
| Funding | 22 | Give the source of funding and the role of the funders for the present study and, if applicable, for the original study on which the present article is based | Page 12 | This study was supported by grants from the Zhongshan Social Welfare and Basic Research Project (2022B1062). The funders had no role in study design, data collection and analysis,decision to publish, or preparation of the manuscript. |

*Give information separately for cases and controls in case-control studies and, if applicable, for exposed and unexposed groups in cohort and cross-sectional studies.

**Note:** An Explanation and Elaboration article discusses each checklist item and gives methodological background and published examples of transparent reporting. The STROBE checklist is best used in conjunction with this article (freely available on the Web sites of PLoS Medicine at http://www.plosmedicine.org/, Annals of Internal Medicine at http://www.annals.org/, and Epidemiology at http://www.epidem.com/). Information on the STROBE Initiative is available at www.strobe-statement.org.
